# Secular trends of dental caries and association with nutritional status: a retrospective analysis of 16,199 Chinese students from three successive national surveys from 2010 to 2019

**DOI:** 10.3389/fpubh.2024.1379767

**Published:** 2024-05-22

**Authors:** Changfu Hao, Yudan Hao, Xiaomin Lou, Xiaolin Wang, Weiping Liu, Huijun Zhou, Xiaolei Ban, Wencan Wu, Xuejie Wang, Xian Wang

**Affiliations:** ^1^College of Public Health, Zhengzhou University, Zhengzhou, China; ^2^Department of Pediatrics, Luohe Central Hospital, Luohe, China; ^3^Department of Child Rehabilitation, The Third Affiliated Hospital of Zhengzhou University, Zhengzhou, China

**Keywords:** caries prevalence, dental caries, nutrition status, body mass index, anemia, children, adolescents

## Abstract

**Introduction:**

The prevalence of dental caries (DC) among students in developing countries has increased at an alarming rate, and nutritional status has been shown to be associated with DC in children and adolescents with inconsistent conclusions. We aimed to understand the trends of DC prevalence in students aged 7, 9, 12, and 14 years and to explore the relationship between DC prevalence and nutritional status.

**Methods:**

We recruited 16,199 students aged 7, 9, 12, and 14 years in China by multi-stage, stratified, random sampling methods from 2010 to 2019. Permanent caries were measured using the Decay, Loss, and Filling (DMF) index and prevalence rate. Deciduous caries were measured using the decay, loss, and filling (dmf) index and prevalence rate. Nutritional status was assessed using body mass index (BMI) and hemoglobin levels. Logistic regression analysis was used to assess the association between nutritional status and the DC prevalence in children and adolescents, incorporating information concerning family-related factors.

**Results:**

The results indicated that DC prevalence increased from 39.75% in 2010 to 53.21% in 2019 in Henan province, with deciduous teeth and permanent teeth being 45.96 and 27.18%, respectively, in 2019. The total caries rate decreased with age (*p* < 0.05), and the caries rate of girls was higher than that of boys in 2019 (55.75% vs. 50.67%) (*p* < 0.001). The prevalence of dental caries among primary and secondary school students in areas with medium economic aggregate was the highest, followed by cities with the best economic development level, and cities with low economic levels have a lower prevalence of dental caries. The dental caries prevalence was negatively correlated with body mass index. In the fully adjusted model, underweight children had a higher caries prevalence (*OR* = 1.10, 95%*CI:* 0.86–1.41). Children with anemia had a higher prevalence of dental caries (*OR* = 1.18, 95%*CI:* 0.98–1.42).

**Conclusion:**

The DC prevalence of students in Henan Province was high, with a tendency to increase. Females, young individuals, and those with a higher economic level showed a positive correlation with the prevalence of caries. In the process of economic development, particular attention should be paid to early childhood caries prevention. Nutritional status should be taken seriously among children and adolescents, and the oral health system should be improved to keep pace with economic development.

## 1 Introduction

Oral diseases affect 3.5 billion people all over the world, and untreated dental caries is the most prevalent condition, affecting nearly 2.4 billion people globally (1, 2). Dental caries is the local destruction of susceptible dental hard tissues by acidic by-products from bacteria fermentation of dietary carbohydrates (3). A meta-analysis from Africa in 2022 showed that the prevalence of caries in permanent teeth among 12-year-olds was 36%, and it continues to rise (4). Another study found that the prevalence of caries among 12 to 14-year-olds in Zhejiang Province in China was 44%. At the same time, the prevalence of caries increased by 3% per year with increasing age (5). Caries has seriously affected the quality of life across all age groups and has caused difficulties in various aspects of life (6, 7). Despite significant advancements in understanding the pathogenesis and etiology of dental caries in recent years, the enduring global economic burden of this disease persists, indicating a failure within the field of dentistry to address its fundamental causes (8, 9).

Dental caries has long been considered a childhood disease. In fact, it persists into adulthood, and health inequalities exist in the burden of dental caries between children and adults (3). A study of adult dental caries from Sweden showed that the side effects of dental caries exist even at a younger age (10). Dental caries in childhood is an overlooked but serious problem (3). Children with tooth decay may experience difficulties in life and learning that can affect their quality of life. These difficulties may include slow eating and sleep disorders (11). Dental caries in children is a dynamic and complex disease process that needs more attention urgently (12). Children aged 7 and 9 years may be experiencing the loss of primary teeth and the eruption of permanent teeth, while children aged 12 and 14 years may already have most of their permanent teeth. Children in this age group are undergoing a critical phase of transitioning from primary teeth to permanent teeth, which significantly impacts their oral health and overall development. This period also marks the establishment of daily oral hygiene routines for children. Through dental caries screening, parents and dentists can educate children about proper brushing and oral care methods to help them establish good oral hygiene habits.

The economic levels in different regions had varying impacts on the prevalence of dental caries. In the past 30 years, the prevalence of dental caries varied greatly between and within populations (13). The burden of oral diseases in impoverished areas has not been accurately understood, and oral diseases affect marginalized populations at varying levels (1, 14). A cross-sectional study showed that the prevalence of dental caries in rural communities was higher than that in urban communities (15). A recent extensive cross-sectional study revealed that the population-attributable proportion of household income has a greater impact on the condition of both deciduous and permanent teeth compared to previously recognized individual risk factors and behaviors (16).

Additionally, there is evidence of a relationship between nutritional status and dental caries (17–19). Moreover, while BMI has been linked to dental caries, the findings in current research have been inconsistent. A Chinese cross-sectional study showed that BMI was negatively correlated with dental caries among children and adolescents in Huizhou (18). However, a study in Saudi showed that children who often suffered from dental caries were more likely to be obese than those who rarely suffered from dental caries (20). Whether there is a link between poor oral health and high BMI is less clear and needs further research (21). Second, several studies have shown that early caries (ECC) in children were associated with anemia. For example, an ecologic study showed a negative correlation between anemia and dental caries in 0–2 and 3–5 year preschoolers (17). A recent meta-analysis showed that iron deficiency anemia was more common in children with ECC (22). BMI and anemia, as important indicators of nutritional status, have always been areas of concern. However, there is a dearth of studies that used these two measures to comprehensively assess the association between nutritional status and dental caries, especially among children and adolescents.

In short, the literature suggests that the association between dental caries and income/socioeconomic status/nutritional status has disparate outcomes, which may depend on the progress of the nutritional transition in different regions and countries. Therefore, we aimed to describe the latest prevalence of dental caries among children and adolescents in 2019 and the trend of DC prevalence over the past 9 years, explore the factors related to dental caries, and evaluate the association between dental caries and nutritional status (BMI and anemia).

## 2 Materials and methods

### 2.1 Data sources

Data were extracted from Henan Province of the Chinese National Survey on Students’ Constitution and Health (CNSSCH) conducted in 2010, 2014, and 2019. The CNSSCH is a series of complex multi-stage, cross-sectional, nationwide surveys on the physical fitness and health status of students in China. At each survey year, the participants were primary and secondary students aged 7–18 years who were selected from the same areas in each province. This study was approved by the Life Science Ethics Review Committee of Zhengzhou University (register number: ZZUIRB2021-56). All participants and/or their parents/guardians provided written informed consent.

### 2.2 Sampling and study population

A multi-stage stratified random cluster sampling method was employed to select three municipalities in Henan Province based on their socioeconomic development levels in 2010 and 2014. These municipalities included Zhengzhou (upper economic level), Xinxiang (moderate economic level), and Zhoukou (lower economic level). It should be pointed out that Jiyuan was added in 2019. The reason was that Jiyuan is a county-level city under the jurisdiction of Henan Province, which has developed rapidly in recent years at a moderate economic level. Primary and secondary schools were selected randomly according to urban and rural areas. Random cluster sampling was carried out based on teaching classes, forming the survey samples. All students aged 7–18 years in the selected classes were invited to participate in the survey.

**Figure 1:**
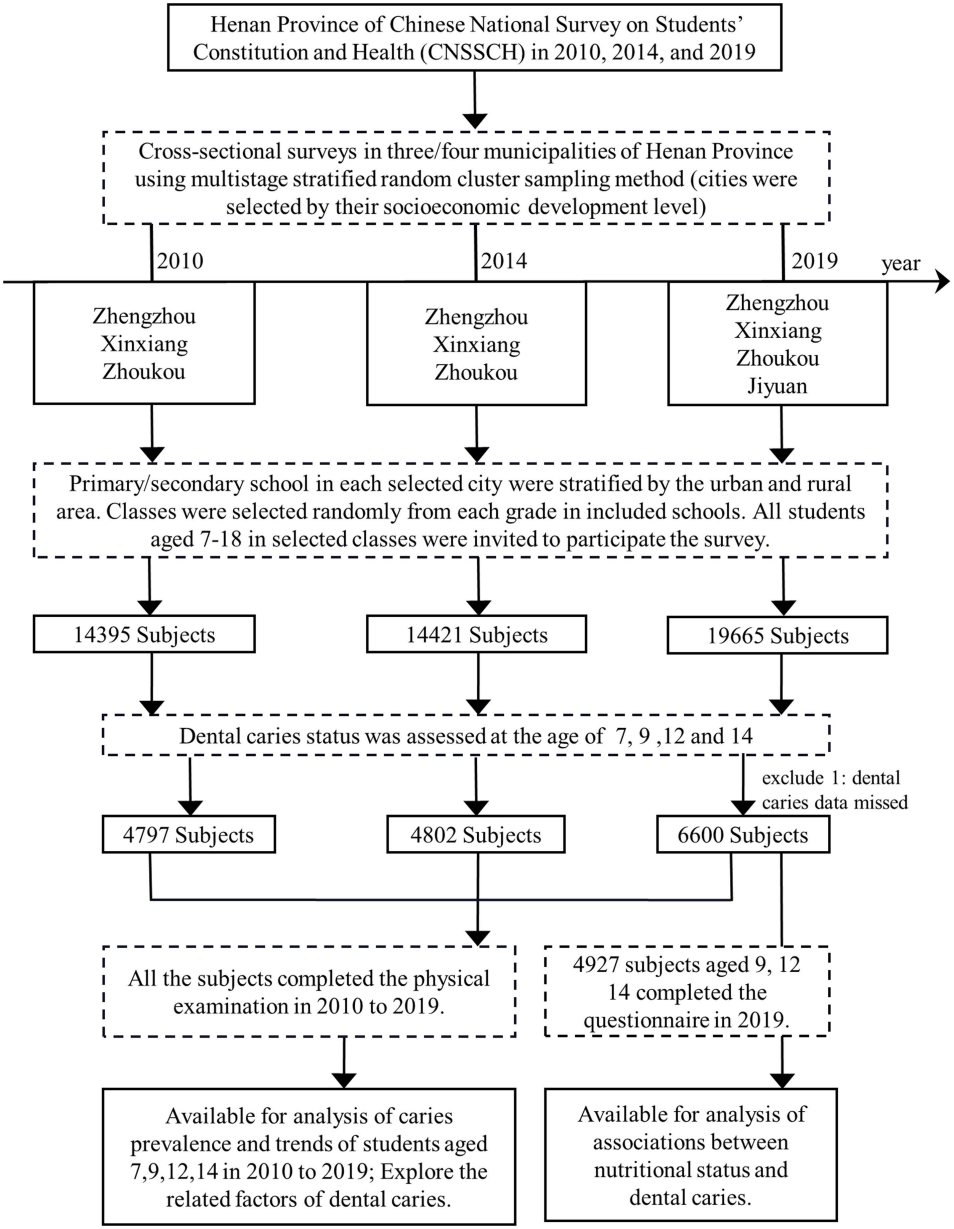
Study flowchart. The study population was selected from the Henan Province of CNSSCH from 2010 to 2019. Three cities in Henan Province were selected according to economic level by multi-stage stratified random cluster sampling method, and Jiyuan was added in 2019. Primary/secondary schools in each selected city were stratified by urban and rural areas and randomly selected classes. All students aged 7–18 years were invited to take part in the survey. A total of 48,481 Han children aged 7–18 years in Henan Province were included in three consecutive cross-sectional surveys, and a total of 4,797 students aged 9, 12, and 14 years completed questionnaire surveys in 2019. CNSSCH, Chinese National Survey on Students’ Constitution and Health.

In this study, a total of 48,481 Han Chinese children aged 7–18 years in Henan province from three consecutive cross-sectional surveys were included from 2010 to 2019, with 14,395 participants in 2010, 14,421 in 2014, and 19,665 in 2019. We tested the students on the corresponding variables, including basic demographic information (age, gender, area, and city) and routine physical examination (height, weight, and hemoglobin). A total of 16,200 students aged 7, 9, 12, and 14 years were examined for caries status from 2010 to 2019, and one subject was excluded due to the missing caries information in 2019. All 16,199 subjects completed the basic demographic information, physical examination, and caries examination, including 4,797 participants in 2010, 4,802 in 2014, and 6,600 in 2019. In addition, a total of 4,927 students aged 9, 12, and 14 years completed questionnaire surveys (including parents’ educational attainment and only child statement) in 2019 (Figure 1).

### 2.3 Examination of dental caries

Caries checks were carried out by trained and qualified staff. Dental caries was examined tooth-by-tooth in quadrantal order for pit, fissure, and groove. All cases of dental caries were defined according to the diagnostic criteria given below: 1. Caries-free: Each tooth showed no signs of caries and had not been filled for caries. 2. Caries: Lesions on the furrow or smooth surface of a tooth with softening of the underside, potential damage to the enamel, or softening of the wall. Caries can be divided into fossa caries and smooth caries (caries on the adjacent, buccal, and lingual surfaces of the teeth). It also includes temporary fillers (such as zinc oxide) on the teeth. The deciduous tooth caries was recorded as “d” and the permanent tooth caries was recorded as “D.” Most notably, primary or secondary caries did not need to be distinguished. For filled teeth with caries, we recorded them as caries. 3. Deciduous tooth lost to decay before it reaches replacement age as “m” and permanent teeth that were lost or removed due to caries as “M.” 4. When there was one or more fillers, and no caries or secondary caries, the deciduous tooth caries was recorded as “f” and the permanent tooth caries was recorded as “M.” The number of decayed, filled, and missed teeth were summed together to give the DMFT score for primary and permanent dentition. The DMF Index and dmf Index for the children were calculated by the following formula:


DMFIndex=D+M+F/Nofparticipants.

dmfIndex=d+m+f/Nofparticipants.

The prevalence of dental caries=Nof dental caries/Nof participants.

### 2.4 Measurements and definition of nutritional status

#### 2.4.1 BMI classification

Height was tested by medical health professionals. Before measurement, we checked 0 points, whether the column was vertical, whether there was shaking, and corrected them in time. Participants stood barefoot on the plate of the height meter their back to the column, ensuring that their torso was naturally straight and their eyes were looking straight ahead. This position ensured that the upper edge of the ear screen was horizontal with the lowest point of the lower edge of the eye socket. Data were recorded accurately, and the error was no more than 0.5 cm. Body weight was measured using an electronic weight meter. Before using it, working conditions, accuracy, and sensitivity were checked to ensure the quality of the measurement. The subject was barefoot and naturally stood in the center of the plate to keep the body stable. After the value was displayed on the screen stably, the tester recorded it in kilograms.

BMI was calculated by weight (kg)/height (m)^2^. All subjects were classified into three nutritional status groups (underweight, normal, and overweight and obesity) according to the screening standard for malnutrition in children and adolescents (WS/T 456–2014) and the screening for overweight and obesity in children and adolescents (WS/T 586–2018) (23). Boys and girls were divided into four age groups (7, 9, 12, and 14 years), and different cutoff values were determined.

#### 2.4.2 Anemia status

Hemoglobin concentration was completed by professional inspectors with disposable blood sampling needles, disinfection supplies, and a HemoCue201+ hemoglobin instrument. After the automatic self-check of the instrument (approximately 10 s), three flashing short lines were displayed on the screen, indicating that the machine was ready for detection. The fingertip to be measured was disinfected, and a blood sampling needle was used to gently puncture the skin on the outside of the fingertip. Two drops of blood were squeezed out and wiped off with a cotton swab. A drop of blood (approximately 10–20 μL) was then gently squeezed from the far end of the finger toward the fingertip. At the same time, several cuvettes were taken, and the blood samples were collected within 10 min. The blood samples were placed inside the analyzer, and the hemoglobin values were displayed on the screen for approximately 15–60 s. The data were recorded accurately.

Anemia was defined by hemoglobin levels. Students aged 7 and 9 years with hemoglobin levels <115 grams per liter and students aged 12 and 14 years with hemoglobin levels <120 grams per liter were considered anemic as proposed by the WHO criteria of hemoglobin screening for children and adolescents (24–26).

### 2.5 Data analysis

The data were analyzed using IBM SPSS version 21.0 (IBM). Continuous variables were characterized as mean ± standard deviation, and categorical variables were characterized by frequencies and percentages. Chi-square tests were used to compare the prevalence of dental caries among children in different groups and explore its associations with related factors. In addition, we stratified BMI and searched for associations with nutritional status in students aged 7, 9, 12, and 14 years. Logistic regression models were used to examine the association between dental caries and nutritional status in students aged 7, 9, 12, and 14 years. Binary logistic regression analysis was adjusted for three different levels of variables: Model 1 adjusted two variables, namely age and gender. Model 2 additionally adjusted for the area and cities of different economic levels. The relationship was quantified as odds ratios (*ORs*). A *p*-value of less than 0.05 was defined as statistically significant.

## 3 Results

### 3.1 DC prevalence of students in Henan Province in 2019

The caries rate of deciduous teeth in students aged 7, 9, and 12 years was 45.96% in 2019. Supplementary Table S1 shows the comparison of caries prevalence in deciduous teeth of students aged 7, 9, and 12 years in Henan Province in 2019. Among the 4,941 screened students, the proportion of boys and girls was similar (49.8% vs. 50.2%, respectively). In addition, the number of students in both areas (urban area and rural area) and in the selected cities (Zhengzhou, Xinxiang, Zhoukou, and Jiyuan) were close. Most of the students were enrolled in the group without anemia (89.6%) compared to anemia (10.4%). With regard to BMI classification, 1,508 participants (64.1%) were in the normal weight group, 1,509 participants (30.5%) were in the overweight and obese group, and only 266 participants (5.4%) were in the underweight group. As shown in Supplementary Table S1, students who were underweight were observed to have a higher prevalence of dental caries in deciduous teeth compared to the normal weight children; however, students who were overweight and obese showed a lower prevalence (*p* < 0.01).

The caries rate of permanent teeth in students aged 7, 9, 12, and 14 years was 27.18% in 2019. Supplementary Table S2 shows the comparison of caries prevalence in permanent teeth of 6,600 students aged 7, 9, 12, and 14 years in Henan Province in 2019. Similar demographic patterns were observed for the 6,600 students. The prevalence of underweight, normal weight, and overweight and obesity was 5.3, 65.5, and 29.2%, respectively. Female students showed a significantly higher prevalence (30.79%) of dental caries in permanent teeth (*p* < 0.01) compared to male students (23.6%). Prevalence of dental caries in permanent teeth increased with increasing age. The 14-year-old students had the highest prevalence of dental caries in permanent teeth (37.6%). In addition, the caries rate of permanent teeth in rural areas was higher than that in urban areas (*p* < 0.01).

**Figure 2:**
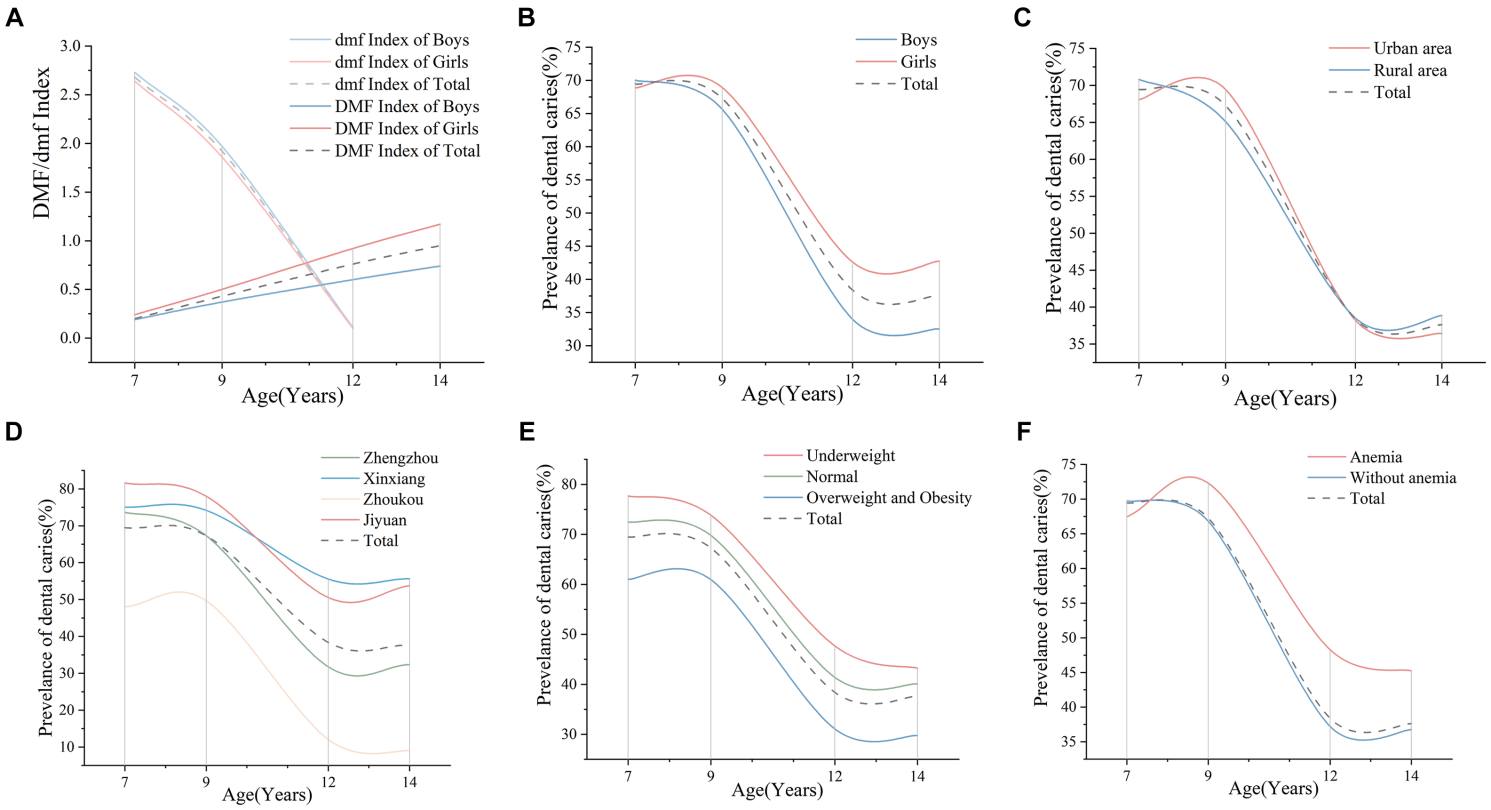
Prevalence of students aged 7, 9, 12, and 14 years with dental caries in 2019, by age group. **(A)** DMF/dmf Index of boys and girls aged 7, 9, 12, and 14 years in school in 2019, by age group. **(B)** Prevalence of dental caries in boys and girls aged 7, 9, 12, and 14 years in 2019, by age group. **(C)** Prevalence of dental caries of students aged 7, 9, 12, and 14 years in urban and rural areas in 2019 by age group. **(D)** Prevalence of dental caries of students aged 7, 9, 12, and 14 years in different cities where the children live in 2019, by age group. **(E)** Prevalence of dental caries in students aged 7, 9, 12, and 14 years with different BMI classifications in 2019. **(F)** Prevalence of dental caries in students aged 7, 9, 12, and 14 years with or without anemia in 2019. DMF, decayed, missing, and filled of permanent teeth; dmft, decayed, missing, and filled of deciduous teeth; BMI, body mass index.

In addition, the DMF Index of deciduous teeth and permanent teeth was described by stratifying age and gender. As shown in Figure 2A, girls showed a sharp increase in the caries rate of permanent teeth with age, particularly in those older than 9 years, whereas boys showed a more stable and smaller increase in the caries rate of permanent teeth with age (boys vs. girls: 1.62 vs. 1.54). Deciduous teeth were just the opposite. As age increased, the DMF index of boys was higher than that of girls, and the rate of caries decreased faster than in girls (boys vs. girls: 0.47 vs. 0.70).

In 2019, the DC prevalence of students in Henan Province was 53.21%. Supplementary Table S3 shows the caries prevalence of 6,600 students aged 7, 9, 12, and 14 years stratified by BMI status in Henan Province in 2019 and mentions the risk factors associated with caries status. Data were classified into three BMI parts (underweight, normal weight, and overweight and obese). Overall, the prevalence of DC decreased with increasing BMI in 2019 (*p* < 0.001). We found female, younger, and anemic students had a higher incidence of caries.

As shown in Figure 2B, female students (55.75%) had a higher prevalence of caries than male students (50.67%) (*p* < 0.01). For students in rural areas, the prevalence of caries was higher than that in urban areas. However, the 9-year-old children were on the contrary (Figure 2C). The influence on the cities was found to be statistically significant (*p* < 0.01). Concretely, the prevalence of DC showed a downward trend in the following order: Jiyuan (65.71%), Xinxiang (65.08%), Zhengzhou (51.28%), and Zhoukou (30.14%) (Figure 2D). Jiyuan and Xinxiang, as cities with medium economic levels, had the first and second-highest caries rates. As the city with the highest level of economic development, the prevalence of caries among primary and secondary school students in Zhengzhou was in the middle level, while Zhoukou, with the lowest level of development, had the lowest prevalence of caries.

Prevalence of dental caries in students aged 7, 9, 12, and 14 years with different nutritional status is shown in Figures 2E,F. Interestingly, we found that students who were underweight had a higher DC prevalence (62.82%), while students with a higher BMI tended to have a lower prevalence of caries (Figure 2E). For anemic status, there was a significant difference among students aged 12 years and older (*p* < 0.05). The results suggested that students with anemia had a higher prevalence (58.04%) of caries than students without anemia (52.65%) (Figure 2F).

### 3.2 Trends of dental caries from 2010 to 2019

**Figure 3:**
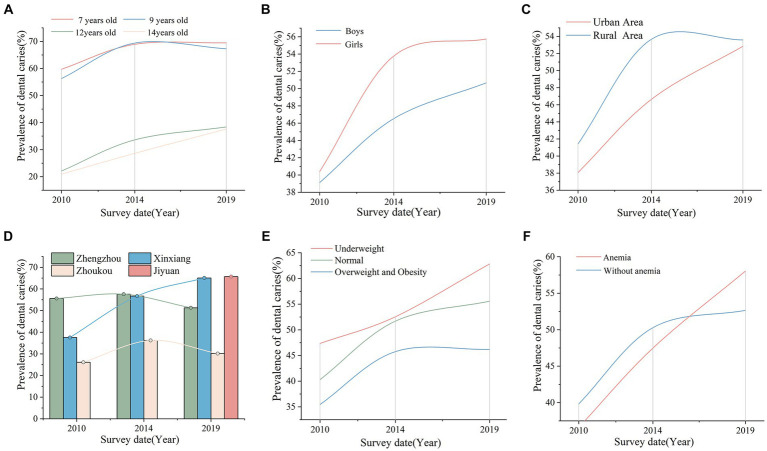
DC prevalence trends of students aged 7, 9, 12, and 14 years in different subgroups from 2010 to 2019. **(A)** Trends of the DC prevalence in students aged 7, 9, 12, and 14 years from 2010 to 2019. **(B)** Trends of the DC prevalence in students of different genders from 2010 to 2019. **(C)** Trends of the DC prevalence in students of different areas from 2010 to 2019. **(D)** Trends of the DC prevalence in students of different cities from 2010 to 2019. **(E)** Trends of the DC prevalence in students of different BMI classifications from 2010 to 2019. **(F)** Trends of the DC prevalence in students of different anemia status from 2010 to 2019. DC, dental caries; BMI, body mass index.

From 2010 to 2019, trends of DC prevalence showed an overall increase from 39.75 to 53.21%. The prevalence of caries decreases with age, flattening out at the age of 12 years and older. However, the prevalence of caries increases as time passes (Figure 3A). Furthermore, the DC prevalence increased significantly over the years in both boys and girls, especially between 2010 and 2014, and remained stable thereafter. For boys, the DC prevalence was 39.12, 46.52, and 50.67% in 2010, 2014, and 2019, respectively, and for girls 40.39, 53.77, and 55.75%, respectively (Figure 3B).

As shown in Supplementary Table S4, the difference between rural areas and urban areas was significant only in 2014 (*p* < 0.01). Not surprisingly, the prevalence of caries among rural children (53.66%) was higher than that among urban children (46.63%) and showed an increasing trend, respectively (Figure 3C). The economic level of cities also had significant differences in dental caries. The DC prevalence in Xinxiang showed an increasing trend; however, Zhengzhou and Zhoukou showed an increasing trend and then a decreasing trend (Figure 3D).

As shown in Figure 3E, overweight individuals had fewer caries from 2010 to 2019 (*p* < 0.001). In 2019, the prevalence of DC was 46.19% in overweight and obese adolescents, 55.57% in normal-weight children, and 62.82% in underweight students. In addition, we found that students with anemia tended to have higher rates of caries. From 2010 to 2019, the prevalence of DC in anemic children ranged from 36.61 to 58.04%, and in non-anemic students, it ranged from 39.83 to 52.65% (Figure 3F).

### 3.3 Associations between dental caries and nutritional status in 2019

Supplementary Table S4 shows the associations between dental caries and nutritional status among students aged 7, 9, 12, and 14 years in Henan Province in 2019. We found underweight was a risk factor (crude *OR* = 1.35, 95%*CI*: 1.08, 1.69) for dental caries, and obesity was a protective factor (crude *OR* = 0.69, 95%*CI*: 0.62, 0.76) for dental caries. Some variables were adjusted using logistic regression, and the result was consistent. Model 1 adjusted two variables, namely age and gender; Model 2 additionally adjusted the areas and economic level of cities. When compared to the normal-weight students, DC prevalence in overweight and obese students was lower (*OR* = 0.72, 95%*CI*: 0.64, 0.81).

## 4 Discussion

The study revealed that in 2019, the prevalence of dental caries among children from Henan was 53.21%, suggesting a persistently high prevalence of caries in this population. From 2010 to 2019, there was an increasing trend for students in Henan Province. Therefore, the families and government should pay more attention to the effects of DC on children and initiate early interventions on factors fueling DC (27–29). First, it is of paramount importance to educate children about the correct brushing techniques and methods (30). Second, it is important to manage dietary habits effectively (31). This entails reducing sugar and refined carbohydrate intake and avoiding eating before bed. It is also beneficial to encourage a healthy diet, including foods rich in calcium and vitamin D, to promote healthy tooth development. Third, it is crucial to undergo regular dental check-ups. These should be carried out by dental professionals on a regular basis to enable early detection of cavities and other oral problems (32). Fourth, in the event that caries has already manifested, treatment should be initiated as expeditiously as possible to forestall further deterioration of the dental structure. This may be accomplished through the use of non-invasive techniques, such as the application of fluoride gels or coatings. In the case of cavities that have already formed, dental restoration is indicated. Such treatments may include filling, root canal, or tooth extraction, if necessary. Following treatment, periodic check-ups are performed to monitor the status of the teeth, and prevention and screening plans are adjusted according to the effectiveness of treatment and the child’s oral health. Finally, it is imperative that policy efforts be made to limit the sale of foods and beverages that are known to cause dental caries (33). In most developed countries, the incidence rate of dental caries has declined in the past 20 years. In contrast, the prevalence of dental caries has been increasing in many developing countries (34). China is the most populous developing country in the world, but it has developed rapidly, and the development of various regions is uneven. In other words, there are significant disparities in terms of economy, education, medical level, and other factors among different regions in China. As a result, the prevalence of oral diseases varies from province to province (5).

In the study population, the prevalence of permanent tooth decay was 30.79% in girls and 23.56% in boys. There was a positive correlation between gender and the prevalence of permanent tooth caries, which is in line with findings from other studies (35, 36). This phenomenon may be attributed to the accelerated growth and development of girls, resulting in earlier eruption of permanent teeth. Consequently, girls are exposed to cariogenic factors for an extended duration, thereby contributing to a higher prevalence of dental caries (37). In addition, dental caries is related to snacks and sweet foods. Several studies showed that girls preferred sweet foods (38, 39), which may also lead to the gender difference in permanent tooth caries in adolescents. On the other hand, age plays an important role in affecting the caries rate of children and adolescents (12). In this study, with the increase of age, permanent tooth caries increased correspondingly, while the prevalence of deciduous tooth caries decreased. Several reports have shown that the association between age and dental caries was estimated as being more prominent in older adult children over 12 years of age (40) in that all deciduous teeth of children were replaced by permanent teeth at approximately 12 years old. As children grow older, the deciduous teeth gradually fall out, and the number of permanent teeth increases. At the same time, the cumulative exposure time of the permanent teeth becomes longer, potentially increasing the likelihood of caries (41). Therefore, the caries rate of deciduous teeth decreased, and the caries rate of permanent teeth increased with age.

Henan, located in central China, is typical of a rural province with a large rural population and a moderately developed economy. Approximately 79% of the population resides in rural areas (42). Most studies have indicated that the prevalence of caries is higher in rural areas than in urban areas. A cross-sectional study involving 963 children in south-eastern Louisiana yielded similar results (15). It is noteworthy that the caries rate of permanent teeth we found in Henan rural areas was higher than that in urban areas. However, this association was not observed in deciduous teeth in our study. This discrepancy may largely be attributed to socioeconomic inequality, although it is not the sole factor influencing the results (43). Surprisingly, in addition to urban–rural differences, caries prevalence varied significantly among different regions of Henan Province. Specifically, the prevalence of dental caries among primary and secondary school students in areas with medium economic aggregate was the highest, followed by cities with the best economic development level, and cities with low economic levels had a lower prevalence of dental caries. This may be due to the fact that teenagers in areas with a high economic aggregate have the opportunity to eat sweets and drinks more frequently, leading to an increased risk of developing caries (16). However, areas with higher economic development levels may pay more attention to health education, such as frequent oral health education, to improve awareness of oral health among students, which may be the reason for the slow decline in the prevalence of dental caries in developed countries. Surprisingly, caries in children and adolescents was most common in developing countries with middle and high income (44). Although these countries were becoming more urbanized, caries-causing foods were becoming more plentiful, and dental services were not improving at a consistent pace. With the continuous development of the economy, society should pay more attention to preventive measures for oral health. Furthermore, policy and practice require a better understanding of how economic development aligns with the oral health system. Further implications of policy and practice require a better understanding of how economic development matches with oral health systems in addition to the impact of poverty.

For nutritional status, the prevalence of overweight and obesity was 29.2%, and the prevalence of anemia was 10.4%. First, with the increase in BMI levels, the prevalence of dental caries decreased. This trend still existed in the subgroups of gender, age, urban and rural, and in the cities at different economic levels. There was a negative correlation between BMI classification and caries after adjusting for confounding factors. The result was consistent with some previous studies (18, 44). The reason may be that family and school intervened in the living habits of obese adolescents (45), such as reducing the intake of sweets and playing a role in promoting the protection of teeth to a certain extent. However, it was also possible that untreated, permanent tooth caries may lead to decreased appetite (46), eating pain, and reduced food consumption, resulting in a lower BMI. Several studies have shown inconsistent results (20, 21, 47). The causal relationship needs to be further demonstrated. In addition, we found children with anemia were more likely to suffer from dental caries compared to those without anemia. A systematic review and meta-analysis showed anemia was more prevalent in children with early childhood caries (22). This is consistent with our research results.

The large and representative sample was one of the strengths of the present study. The results provided a theoretical basis for further intervention of caries in children and adolescents. However, there were several limitations to consider. First of all, this study did not collect the oral health habits of each teenager, and further research on the relationship between oral health habits and dental caries needs to be carried out. Second, the timing relationship between exposure and outcome cannot be determined, and there may be an impact of deciduous tooth caries on the formation of permanent tooth caries. In addition, sociodemographic factors are not widely collected and should be valued and included in the future. Finally, the research subjects were from junior and senior high schools in Henan Province. It may be inaccurate to extrapolate the research results to other regions because regional influence and economic levels fluctuate greatly.

## 5 Conclusion

Three consecutive cross-sectional studies have demonstrated a positive correlation between the prevalence of dental caries and being female, young, and economically advantaged. Conversely, good nutritional status has shown a negative association with the prevalence of dental caries. In light of these findings, optimizing nutritional status, providing timely treatment for anemia, and enhancing oral health education efforts may serve as practical strategies to mitigate the rapidly increasing rates of dental caries among children and adolescents in developing countries.

## Data availability statement

The original contributions presented in the study are included in the article/Supplementary material, further inquiries can be directed to the corresponding author.

## Ethics statement

The studies involving humans were approved by the Life Science Ethics Review Committee of Zhengzhou University. The studies were conducted in accordance with the local legislation and institutional requirements. Written informed consent for participation in this study was provided by the participants’ legal guardians/next of kin.

## Author contributions

CH: Conceptualization, Investigation, Software, Writing – original draft, Data curation, Validation. YH: Conceptualization, Supervision, Formal analysis, Methodology, Visualization, Writing – original draft. XL: Project administration, Supervision, Writing – review & editing. XOW: Data curation, Investigation, Software, Writing – review & editing. WL: Data curation, Investigation, Software, Writing – review & editing. HZ: Data curation, Formal analysis, Validation, Writing – review & editing. XB: Data curation, Formal analysis, Validation, Writing – review & editing. WW: Data curation, Formal analysis, Validation, Writing – review & editing. XuW: Data curation, Formal analysis, Validation, Writing – review & editing. XiW: Conceptualization, Funding acquisition, Project administration, Resources, Supervision, Writing – review & editing.
