# Supplementary material for: Secular trends of dental caries and association with nutritional status: a retrospective analysis of 16,199 Chinese students from three successive national surveys from 2010 to 2019
Source: Front Public Health. 2024 May 22;12:1379767. doi: 10.3389/fpubh.2024.1379767 (PMC11150691; doi:10.3389/fpubh.2024.1379767)
Supplement: Supplementary file 1 [file Data_Sheet_1.doc]

Table S1. Comparison of caries prevalence in deciduous teeth of students aged 7, 9, 12 in 2019

| **Age(years)/**  **Subgroup** | **7** | | |  | **9** | | |  | **12** | | |  | **Total** | | |
| --- | --- | --- | --- | --- | --- | --- | --- | --- | --- | --- | --- | --- | --- | --- | --- |
|  | Study participants | Caries(N,%) | *P*-value |  | Study participants | Caries(N,%) | *P*-value |  | Study participants | Caries(N,%) | *P*-value |  | Study participants | Caries(N,%) | *P*-value |
| **Gender** |  |  | 0.696 |  |  |  | 0.614 |  |  |  | 0.489 |  |  |  | 0.613 |
| Male | 831 | 567(68.23) |  |  | 827 | 515(62.27) |  |  | 803 | 58(7.22) |  |  | 2461 | 1140(46.32) |  |
| Female | 842 | 567(67.34) |  |  | 805 | 511(63.48) |  |  | 833 | 53(6.36) |  |  | 2480 | 1131(45.60) |  |
| **Area** |  |  | 0.865 |  |  |  | 0.020 |  |  |  | 0.138 |  |  |  | 0.354 |
| Urban area | 833 | 563(67.59) |  |  | 815 | 535(65.64) |  |  | 847 | 65(7.67) |  |  | 2495 | 1163(46.61) |  |
| Rural area | 840 | 571(67.98) |  |  | 817 | 491(60.10) |  |  | 789 | 46(5.83) |  |  | 2446 | 1108(45.3) |  |
| **City** |  |  | <0.001 |  |  |  | <0.001 |  |  |  | 0.004 |  |  |  | <0.001 |
| Zhengzhou | 402 | 282(70.15) |  |  | 401 | 255(63.59) |  |  | 400 | 22(5.50) |  |  | 1203 | 559(46.47) |  |
| Xinxiang | 413 | 306(74.09) |  |  | 395 | 270(68.35) |  |  | 405 | 30(7.41) |  |  | 1213 | 606(49.96) |  |
| Zhoukou | 429 | 204(47.55) |  |  | 413 | 193(46.73) |  |  | 374 | 14(3.74) |  |  | 1216 | 411(33.80) |  |
| Jiyuan | 429 | 342(79.72) |  |  | 423 | 308(72.81) |  |  | 457 | 45(9.85) |  |  | 1309 | 695(53.09) |  |
| **Anemia** |  |  | 0.460 |  |  |  | 0.169 |  |  |  | 0.484 |  |  |  | 0.652 |
| Yes | 212 | 139(65.57) |  |  | 130 | 89(68.46) |  |  | 174 | 14(8.05) |  |  | 516 | 242(46.90) |  |
| No | 1461 | 995(68.10) |  |  | 1502 | 937(62.38) |  |  | 1462 | 97(6.63) |  |  | 4425 | 2029(45.85) |  |
| **BMI classification** |  |  | <0.001 |  |  |  | 0.001 |  |  |  | 0.001 |  |  |  | <0.001 |
| Underweight | 94 | 72(76.60) |  |  | 107 | 74(69.16) |  |  | 65 | 12(18.46) |  |  | 266 | 158(59.40) |  |
| Normal | 1097 | 775(70.65) |  |  | 1013 | 663(65.45) |  |  | 1056 | 70(6.63) |  |  | 3166 | 1508(47.63) |  |
| Overweight and obesity | 482 | 287(59.54) |  |  | 512 | 289(56.45) |  |  | 515 | 29(5.63) |  |  | 1509 | 605(40.09) |  |
| **Total** | 1673 | 1134(67.78) |  |  | 1632 | 1026(62.87) |  |  | 1636 | 111(6.78) |  |  | 4941 | 2271(45.96) |  |

Table S2. Comparison of caries prevalence in permanent teeth of students aged 7, 9, 12, 14 in 2019

| **Age(years)/**  **Subgroup** | **7** | | |  | **9** | | |  | **12** | | |  | **14** | | |  | **Total** | | |
| --- | --- | --- | --- | --- | --- | --- | --- | --- | --- | --- | --- | --- | --- | --- | --- | --- | --- | --- | --- |
|  | Study participants | Caries(N,%) | *P*-value |  | Study participants | Caries(N,%) | *P*-value |  | Study participants | Caries(N,%) | *P*-value |  | Study participants | Caries(N,%) | *P*-value |  | Study participants | Caries(N,%) | *P*-value |
| **Gender** |  |  | 0.126 |  |  |  | 0.004 |  |  |  | <0.001 |  |  |  | <0.001 |  |  |  | <0.001 |
| Male | 831 | 103(12.39) |  |  | 827 | 164(19.83) |  |  | 803 | 238(29.64) |  |  | 833 | 271(32.53) |  |  | 3294 | 776(23.56) |  |
| Female | 842 | 126(14.96) |  |  | 805 | 208(25.84) |  |  | 833 | 331(39.74) |  |  | 826 | 353(42.74) |  |  | 3306 | 1018(30.79) |  |
| **Area** |  |  | 0.001 |  |  |  | 0.003 |  |  |  | 0.494 |  |  |  | 0.307 |  |  |  | 0.001 |
| Urban area | 833 | 90(10.80) |  |  | 815 | 161(19.75) |  |  | 847 | 288(34.00) |  |  | 843 | 307(36.42) |  |  | 3338 | 846(25.34) |  |
| Rural area | 840 | 139(16.55) |  |  | 817 | 211(25.83) |  |  | 789 | 281(35.61) |  |  | 816 | 317(38.85) |  |  | 3262 | 948(29.06) |  |
| **City** |  |  | <0.001 |  |  |  | <0.001 |  |  |  | <0.001 |  |  |  | <0.001 |  |  |  | <0.001 |
| Zhengzhou | 402 | 59(14.68) |  |  | 401 | 84(20.95) |  |  | 400 | 116(29.00) |  |  | 402 | 130(32.34) |  |  | 1605 | 389(24.24) |  |
| Xinxiang | 413 | 61(14.77) |  |  | 395 | 132(33.42) |  |  | 405 | 213(52.59) |  |  | 408 | 227(55.64) |  |  | 1621 | 633(39.05) |  |
| Zhoukou | 429 | 6(1.40) |  |  | 413 | 17(4.12) |  |  | 374 | 33(8.82) |  |  | 423 | 38(8.98) |  |  | 1639 | 94(5.74) |  |
| Jiyuan | 429 | 103(24.01) |  |  | 423 | 139(32.86) |  |  | 457 | 207(45.30) |  |  | 426 | 229(53.76) |  |  | 1735 | 678(39.08) |  |
| **Anemia** |  |  | 0.834 |  |  |  | 0.463 |  |  |  | 0.003 |  |  |  | 0.031 |  |  |  | 0.005 |
| Yes | 212 | 30(14.15) |  |  | 130 | 33(25.38) |  |  | 174 | 78(44.83) |  |  | 168 | 76(45.24) |  |  | 684 | 217(31.73) |  |
| No | 1461 | 199(13.62) |  |  | 1502 | 339(22.57) |  |  | 1462 | 491(33.58) |  |  | 1491 | 548(36.75) |  |  | 5916 | 1577(26.66) |  |
| **BMI classification** |  |  | 0.165 |  |  |  | 0.062 |  |  |  | <0.001 |  |  |  | 0.001 |  |  |  | <0.001 |
| Underweight | 94 | 13(13.83) |  |  | 107 | 29(27.10) |  |  | 65 | 23(35.38) |  |  | 81 | 35(43.21) |  |  | 347 | 100(28.82) |  |
| Normal | 1097 | 162(14.77) |  |  | 1013 | 244(24.09) |  |  | 1056 | 403(38.16) |  |  | 1158 | 81(40.07) |  |  | 4324 | 1273(29.44) |  |
| Overweight and obesity | 482 | 54(1.12) |  |  | 512 | 99(19.34) |  |  | 515 | 143(27.77) |  |  | 420 | 125(29.76) |  |  | 1929 | 421(21.82) |  |
| **Total** | 1673 | 229(13.69) |  |  | 1632 | 372(22.79) |  |  | 1636 | 569(34.78) |  |  | 1659 | 624(37.61) |  |  | 6600 | 1794(27.18) |  |

Table S3. Caries prevalence of students aged 7, 9, 12, 14 stratified by BMI status in 2010

| BMI Classification/  Subgroup | Underweight | | |  | Normal | | |  | Overweight and obesity | | |  | Total | | |
| --- | --- | --- | --- | --- | --- | --- | --- | --- | --- | --- | --- | --- | --- | --- | --- |
|  | Study participants | Caries(N,%) | *P*-value |  | Study participants | Caries(N,%) | *P*-value |  | Study participants | Caries(N,%) | *P*-value |  | Study participants | Caries(N,%) | *P*-value |
| **Gender** |  |  | 0.773 |  |  |  | 0.841 |  |  |  | 0.04 |  |  |  | 0.367 |
| male | 195 | 91(46.67) |  |  | 1623 | 657(40.48) |  |  | 580 | 190(32.76) |  |  | 2398 | 938(39.12) |  |
| female | 143 | 69(48.25) |  |  | 1801 | 723(40.14) |  |  | 455 | 177(38.90) |  |  | 2399 | 969(40.39) |  |
| **Age** |  |  | <0.001 |  |  |  | <0.001 |  |  |  | <0.001 |  |  |  | <0.001 |
| 7 | 72 | 50(69.44) |  |  | 848 | 521(61.44) |  |  | 279 | 145(51.97) |  |  | 1199 | 716(59.72) |  |
| 9 | 98 | 64(65.31) |  |  | 813 | 472(58.06) |  |  | 289 | 139(48.10) |  |  | 1200 | 675(56.25) |  |
| 12 | 70 | 18(25.71) |  |  | 877 | 205(23.38) |  |  | 252 | 42(16.67) |  |  | 1199 | 265(22.10) |  |
| 14 | 98 | 28(28.57) |  |  | 886 | 182(20.54) |  |  | 215 | 41(19.07) |  |  | 1199 | 251(20.93) |  |
| **Area** |  |  | 0.029 |  |  |  | 0.421 |  |  |  | 0.313 |  |  |  | 0.017 |
| Urban area | 137 | 55(40.15) |  |  | 1547 | 612(39.56) |  |  | 714 | 246(34.45) |  |  | 2398 | 913(38.07) |  |
| Rural area | 201 | 105(52.24) |  |  | 1877 | 768(40.92) |  |  | 321 | 121(37.69) |  |  | 2399 | 994(41.43) |  |
| **City** |  |  | <0.001 |  |  |  | <0.001 |  |  |  | <0.001 |  |  |  | <0.001 |
| Zhengzhou | 125 | 77(61.60) |  |  | 1115 | 630(56.50) |  |  | 359 | 182(50.70) |  |  | 1599 | 889(55.60) |  |
| Xinxiang | 173 | 73(42.20) |  |  | 1131 | 436(38.55) |  |  | 296 | 92(31.08) |  |  | 1600 | 601(37.56) |  |
| Zhoukou | 40 | 10(25.00) |  |  | 1178 | 314(26.66) |  |  | 380 | 93(24.47) |  |  | 1598 | 417(26.10) |  |
| **Anemia** |  |  | 0.568 |  |  |  | 0.210 |  |  |  | 0.417 |  |  |  | 0.491 |
| Yes | 5 | 3(60.00) |  |  | 94 | 32(34.04) |  |  | 13 | 6(46.15) |  |  | 112 | 41(36.61) |  |
| No | 333 | 157(47.15) |  |  | 3330 | 1348(40.48) |  |  | 1022 | 361(35.32) |  |  | 4685 | 1866(39.82) |  |
| **Total** | 338 | 160(47.34) |  |  | 3424 | 1380(40.30) |  |  | 1035 | 367(35.46) |  |  | 4797 | 1907(39.75) |  |

Table S4. Caries prevalence of students aged 7, 9, 12, 14 stratified by BMI status in 2014

| BMI Classification/  Subgroup | Underweight | | |  | Normal | | |  | Overweight and obesity | | |  | Total | | |
| --- | --- | --- | --- | --- | --- | --- | --- | --- | --- | --- | --- | --- | --- | --- | --- |
|  | Study participants | Caries(N,%) | *P*-value |  | Study participants | Caries(N,%) | *P*-value |  | Study participants | Caries(N,%) | *P*-value |  | Study participants | Caries(N,%) | *P*-value |
| **Gender** |  |  | 0.028 |  |  |  | 0.006 |  |  |  | <0.001 |  |  |  | <0.001 |
| male | 158 | 74(46.84) |  |  | 1527 | 750(49.12) |  |  | 716 | 293(40.92) |  |  | 2401 | 1117(46.52) |  |
| female | 118 | 71(60.17) |  |  | 1718 | 927(53.96) |  |  | 565 | 293(51.86) |  |  | 2401 | 1291(53.77) |  |
| **Age** |  |  | <0.001 |  |  |  | <0.001 |  |  |  | <0.001 |  |  |  | <0.001 |
| 7 | 72 | 54(75.00) |  |  | 786 | 558(70.99) |  |  | 342 | 215(62.87) |  |  | 1200 | 827(68.92) |  |
| 9 | 74 | 58(78.38) |  |  | 769 | 561(72.95) |  |  | 359 | 215(59.89) |  |  | 1202 | 834(69.38) |  |
| 12 | 61 | 20(32.79) |  |  | 823 | 297(36.09) |  |  | 315 | 86(27.30) |  |  | 1199 | 403(33.61) |  |
| 14 | 69 | 13(18.84) |  |  | 867 | 261(30.10) |  |  | 265 | 70(26.42) |  |  | 1201 | 344(28.64) |  |
| **Area** |  |  | 0.224 |  |  |  | 0.002 |  |  |  | 0.005 |  |  |  | <0.001 |
| Urban area | 118 | 57(48.31) |  |  | 1468 | 714(48.64) |  |  | 814 | 348(42.75) |  |  | 2400 | 1119(46.63) |  |
| Rural area | 158 | 88(55.70) |  |  | 1777 | 963(54.19) |  |  | 467 | 238(50.96) |  |  | 2402 | 1289(53.66) |  |
| **City** |  |  | 0.005 |  |  |  | <0.001 |  |  |  | <0.001 |  |  |  | <0.001 |
| Zhengzhou | 109 | 56(51.38) |  |  | 1069 | 635(59.40) |  |  | 422 | 230(54.50) |  |  | 1600 | 921(57.56) |  |
| Xinxiang | 105 | 66(62.86) |  |  | 1079 | 637(59.04) |  |  | 416 | 204(49.04) |  |  | 1600 | 907(56.69) |  |
| Zhoukou | 62 | 23(37.10) |  |  | 1097 | 405(36.92) |  |  | 443 | 152(34.31) |  |  | 1602 | 580(36.20) |  |
| **Anemia** |  |  | 0.723 |  |  |  | 0.243 |  |  |  | 0.969 |  |  |  | 0.419 |
| Yes | 14 | 8(57.14) |  |  | 159 | 75(47.17) |  |  | 44 | 20(45.45) |  |  | 217 | 103(47.47) |  |
| No | 262 | 137(52.29) |  |  | 3086 | 1602(51.91) |  |  | 1237 | 566(45.76) |  |  | 4585 | 2305(50.27) |  |
| **Total** | 276 | 145(52.54) |  |  | 3245 | 1677(51.68) |  |  | 1281 | 586(45.75) |  |  | 4802 | 2408(50.15) |  |

Table S5. Association of BMI with dental caries stratified by gender

| Gender |  | Underweight | Normal weight | overweight | *P* value | *Kappa* |
| --- | --- | --- | --- | --- | --- | --- |
| Boys | Caries | 120 | 1104 | 445 | <0.001 | <0.001 |
|  | Non-caries | 78 | 932 | 615 |  |  |
| Girls | Caries | 98 | 1299 | 446 | 0.001 | <0.001 |
|  | Non-caries | 51 | 989 | 423 |  |  |
| Total |  | 347 | 4324 | 1929 | <0.001 | <0.001 |

Table S6. Association of BMI with dental caries stratified by age

| Age |  | Underweight | Normal weight | overweight | *P* value | *Kappa* |
| --- | --- | --- | --- | --- | --- | --- |
| 7 | Caries | 73 | 795 | 294 | <0.001 | <0.001 |
|  | Non-caries | 21 | 302 | 188 |  |  |
| 9 | Caries | 79 | 707 | 312 | 0.001 | <0.001 |
|  | Non-caries | 28 | 306 | 200 |  |  |
| 12 | Caries | 31 | 437 | 160 | <0.001 | <0.001 |
|  | Non-caries | 34 | 619 | 355 |  |  |
| 14 | Caries | 35 | 464 | 125 | 0.001 | <0.001 |
|  | Non-caries | 46 | 694 | 295 |  |  |
| Total |  | 347 | 4324 | 1929 |  | <0.001 |

Table S7. Association of anemia status with dental caries stratified by gender

| Gender |  | Anemia | Without anemia | *P* value | *Kappa* |
| --- | --- | --- | --- | --- | --- |
| Boys | Caries | 140 | 1529 | 0.003 | 0.026 |
|  | Non-caries | 93 | 1532 |  |  |
| Girls | Caries | 257 | 1586 | 0.569 | 0.006 |
|  | Non-caries | 194 | 1269 |  |  |
| Total |  | 684 | 5916 | 0.008 | 0.04 |

Table S8. Association of anemia status with dental caries stratified by age

| Age |  | Anemia | Without anemia | *P* value | *Kappa* |
| --- | --- | --- | --- | --- | --- |
| 7 | Caries | 143 | 1019 | 0.498 | -0.008 |
|  | Non-caries | 69 | 442 |  |  |
| 9 | Caries | 94 | 1004 | 0.203 | 0.012 |
|  | Non-caries | 36 | 498 |  |  |
| 12 | Caries | 84 | 544 | 0.005 | 0.051 |
|  | Non-caries | 90 | 918 |  |  |
| 14 | Caries | 76 | 548 |  | 0.038 |
|  | Non-caries | 92 | 943 | 0.031 |  |
| Total |  | 684 | 5916 |  | 0.019 |
